# Use of Household Cluster Investigations to Identify Factors Associated with Chikungunya Virus Infection and Frequency of Case Reporting in Puerto Rico

**DOI:** 10.1371/journal.pntd.0005075

**Published:** 2016-10-20

**Authors:** Danielle Bloch, Nicole M. Roth, Elba V. Caraballo, Jorge Muñoz-Jordan, Elizabeth Hunsperger, Aidsa Rivera, Janice Pérez-Padilla, Brenda Rivera Garcia, Tyler M. Sharp

**Affiliations:** 1 Dengue Branch, Division of Vector-Borne Diseases, Centers for Disease Control and Prevention, San Juan, Puerto Rico; 2 Department of Epidemiology of Microbial Disease, Yale School of Public Health, New Haven, Connecticut; 3 Rollins School of Public Health, Emory University, Atlanta, Georgia; 4 Puerto Rico Department of Health, San Juan, Puerto Rico; University of Texas Medical Branch, UNITED STATES

## Abstract

**Background:**

Chikungunya virus (CHIKV) is transmitted by *Aedes* species mosquitoes and is the cause of an acute febrile illness characterized by potentially debilitating arthralgia. After emerging in the Caribbean in late 2013, the first locally-acquired case reported to public health authorities in Puerto Rico occurred in May 2014. During June–August 2014, household-based cluster investigations were conducted to identify factors associated with infection, development of disease, and case reporting.

**Methodology/Principal Findings:**

Residents of households within a 50-meter radius of the residence of laboratory-positive chikungunya cases that had been reported to Puerto Rico Department of Health (PRDH) were offered participation in the investigation. Participants provided a serum specimen and answered a questionnaire that collected information on demographic factors, household characteristics, recent illnesses, healthcare seeking behaviors, and clinical diagnoses. Current CHIKV infection was identified by rRT-PCR, and recent CHIKV infection was defined by detection of either anti-CHIKV IgM or IgG antibody. Among 250 participants, 74 (30%) had evidence of CHIKV infection, including 12 (5%) with current and 62 (25%) with recent CHIKV infection. All specimens from patients with CHIKV infection that were collected within four days, two weeks, and three weeks of illness onset were positive by RT-PCR, IgM ELISA, and IgG ELISA, respectively. Reporting an acute illness in the prior three months was strongly associated with CHIKV infection (adjusted odds ratio [aOR] = 21.6, 95% confidence interval [CI]: 9.24–50.3). Use of air conditioning (aOR = 0.50, 95% CI = 0.3–0.9) and citronella candles (aOR = 0.4, 95% CI = 0.1–0.9) were associated with protection from CHIKV infection. Multivariable analysis indicated that arthralgia (aOR = 51.8, 95% CI = 3.8–700.8) and skin rash (aOR = 14.2, 95% CI = 2.4–84.7) were strongly associated with CHIKV infection. Hierarchical cluster analysis of signs and symptoms reported by CHIKV-infected participants demonstrated that fever, arthralgia, myalgia, headache, and chills tended to occur simultaneously. Rate of symptomatic CHIKV infection (defined by arthralgia with fever or skin rash) was 62.5%. Excluding index case-patients, 22 (63%) participants with symptomatic CHIKV infection sought medical care, of which 5 (23%) were diagnosed with chikungunya and 2 (9%) were reported to PRDH.

**Conclusions/Significance:**

This investigation revealed high rates of CHIKV infection among household members and neighbors of chikungunya patients, and that behavioral interventions such as use of air conditioning were associated with prevention of CHIKV infection. Nearly two-thirds of patients with symptomatic CHIKV infection sought medical care, of which less than one-quarter were reportedly diagnosed with chikungunya and one-in-ten were reported to public health authorities. These findings emphasize the need for point-of-care rapid diagnostic tests to optimize identification and reporting of chikungunya patients.

## Author Summary

Chikungunya is a mosquito-borne virus that causes an acute febrile illness that often occurs with severe joint pain. The virus first arrived in the Western Hemisphere in late 2013 and has since caused epidemics in much of the Caribbean and the Americas. During the first months of the 2014 epidemic in Puerto Rico, we conducted household-based cluster investigations to identify factors associated with chikungunya virus infection and progression to disease. We found that using air conditioning and citronella candles in and around the home were associated with decreased rates of infection. Symptoms significantly associated with chikungunya virus infection included fever, joint pain, skin rash, and arthritis. Less than one-quarter of participants infected with chikungunya virus that sought medical care were diagnosed with chikungunya and one-in-ten were reported to public health authorities. This investigation demonstrates the importance of household-level behavioral interventions to avoid chikungunya virus infection, as well as the need for rapid diagnostic tests to improve identification of chikungunya patients.

## Introduction

Chikungunya virus (CHIKV) is a mosquito-transmitted alphavirus that can cause an acute febrile illness characterized by potentially debilitating arthralgia [1]. *Aedes aegypti* and *Ae*. *albopictus* mosquitoes are the most common vectors of CHIKV and also transmit the four viruses that cause dengue (DENV-1–4) [1]. CHIKV previously caused outbreaks in Southeast Asian and African countries where large portions of the population (e.g., 38–75%) were affected [2–5], which may be attributable to high viremia in the host, high viral load in mosquitos, immunologically naive populations, and the absence of sustainable and effective vector control methods [6]. Although infection with CHIKV results in long-term protection from reinfection [7], it has been associated with persistent arthritis and/or arthralgia that may last several months [8, 9]. In areas where both CHIKV and DENVs circulate, misdiagnosis of chikungunya may be common, as patients with either disease may present with fever, myalgia, and arthralgia [10].

The first documented locally-acquired chikungunya case in the Western Hemisphere was reported in December 2013 on the Caribbean island of St. Martin [11]. Soon after, CHIKV spread to at least 45 countries and territories throughout the Americas where over 2 million suspected cases have been reported to date [12]. In the United States territory of Puerto Rico, the first laboratory-confirmed chikungunya case occurred in a patient from the San Juan metropolitan area who had illness onset in May 2014 and no history of recent travel [13]. The peak of cases reported through passive surveillance occurred in August 2014 [14], and to date >30,000 suspected chikungunya cases have been reported [15]. However, detection of anti-CHIKV antibodies in nearly 25% of blood donated during 2014 suggests a higher incidence of infection than was reported to public health authorities [16].

Because they are transmitted by the same mosquito vectors, CHIKV is thought to have similar transmission patterns as DENV, which often results in clusters of infected individuals in and around the households where infected individuals reside [17–20]. This is largely due to the anthropophilic nature of *Ae*. *aegypti*, which tend to disperse relatively short distances (<100 meters) and congregate around households [18]. Consequently, human movement has been identified as the primary mode of DENV dissemination beyond 100 meters [21]. Human population density, particularly in relation to urban centers, has also been associated with clustering of chikungunya cases [22].

Following the introduction of CHIKV into Puerto Rico, we conducted household-based cluster investigations to describe the spectrum of disease and factors associated with CHIKV infection, identify host factors associated with symptomatic infection, describe care-seeking behavior in individuals with chikungunya, and identify patient characteristics associated with accurate clinical diagnosis and case reporting of chikungunya patients.

## Methods

### Ethics statement

The investigation protocol underwent institutional review at CDC and was determined to be public health practice and not research. As such, institutional review board approval was not required.

### Investigation design

Puerto Rico, an unincorporated territory of the United States located in the Caribbean Sea, has an area of 3,424 square miles and in 2014 had an estimated population of 3,548,397 (1,036 residents per square mile) [23]. A cross-sectional investigation was conducted in which neighbors of chikungunya patients were offered enrollment in household-based cluster investigations. A convenience sample of laboratory-positive chikungunya cases was identified from suspected chikungunya cases that were reported to Puerto Rico Department of Health (PRDH) and tested laboratory-positive for CHIKV infection (“index cases”). Index case-patients or their parent or guardian were contacted by telephone within 30 days of the index case-patients’ illness onset and a home visit was scheduled. All household investigations were conducted between June 20 and August 19, 2014 (S1 Fig).

During each household visit, the head-of-household of the index case-patient’s household (the “index household”) and all households within a 50-meter radius of the index household were eligible for enrollment in the investigation. If the head-of-household agreed to participate in the investigation, all available members of the household were offered participation. Households were not revisited if the head-of-household was not home or declined participation. A questionnaire (S1 Appendix) addressing household characteristics was administered to the head-of-household, and an individual questionnaire (S1 Appendix) addressing demographics, travel history, and recent illnesses was administered to all participants. Parents or guardians answered individual questionnaires by proxy for participants aged <8 years.

### Diagnostic testing

Serum specimens were collected from all household investigation participants and transported to CDC Dengue Branch in San Juan, Puerto Rico for diagnostic testing. To detect evidence of CHIKV infection, all specimens were tested by rRT-PCR [24], IgM antibody capture (MAC) ELISA [25], and IgG ELISA [26]. Specimens were also tested for evidence of DENV infection, the results of which have been previously reported [13]. In summary, 5% of participants were positive for recent DENV infection, and none were positive for current DENV infection. Inclusion of DENV diagnostic test results in epidemiologic analyses did not appreciably affect the statistical significance of any findings, as there was minimal overlap of participants with evidence of infection with both CHIKV and DENV (i.e., 1 of 74 [1.4%]). Hence, DENV diagnostic test results are not included in the analyses presented herein.

Names and dates of birth of all CHIKV-infected participants were queried in surveillance databases at CDC and PRDH to determine if they had been reported as a suspected chikungunya case-patient.

### Definitions

*Participants* were individuals that provided a serum specimen and answered an individual questionnaire. *Current CHIKV infection* was defined by detection of CHIKV nucleic acid by rRT-PCR. Because CHIKV was first detected to be circulating in Puerto Rico in May 2014 and all household investigations were completed by mid-August 2014, *recent CHIKV infection* was defined by detection of either anti-CHIKV IgM antibody by MAC ELISA or anti-CHIKV IgG antibody by IgG ELISA. Participants were defined as being *laboratory-positive for CHIKV infection* if they had evidence of either current or recent infection. Participants were defined as *laboratory-negative for CHIKV infection* if they had no evidence of either current or recent CHIKV infection. For participants with current CHIKV infection that did not report any symptom of illness (n = 2), development of illness after interview was ruled out by follow-up phone call within 30 days of the household visit. Findings from multivariable and hierarchical clustering analysis of signs and symptoms associated with current or recent CHIKV infection were used to define *symptomatic CHIKV infection*.

### Statistical analyses

General estimating equations (GEE) were used to model associations between individual health and household characteristics and binary outcomes of CHIKV infection status, correct chikungunya diagnosis, or asymptomatic infection. All GEE models were fit with a logit link and assuming an exchangeable correlation matrix. This method estimates the population-averaged effect, accounting for correlations in data of members from the same household and investigation cluster that might otherwise bias estimates [27]. Multivariate GEE analysis was performed to obtain a final model for the association between laboratory-positivity and symptoms reported among participants with illness in the past three months. Backward elimination was used in best-fitting model selection, removing variables from the full model that lowered the Quasilikelihood Information Criteria (QIC) relative to the full model [28]. Hierarchical cluster analysis, which uses a distance measure to identify similar clusters of variables and an agglomeration method to link clusters, was performed to analyze patterns of symptoms among participants with recent illness. Manhattan distance, a measure of similarity that sums the absolute differences among observations, was used due to the binary nature of outcomes. Ward’s method, which groups variables by minimizing the internal sum of squares, was used as the agglomeration method [29]. GEE analyses were performed using SAS 9.3 (SAS Institute Inc., Cary, NC), and hierarchical cluster analysis was performed using R version 3.2.3. ArcGIS version 10.2 (ESRI, Redlands, CA) was used for mapping household clusters.

## Results

### Identification of CHIKV-infected participants

**Fig 1:**
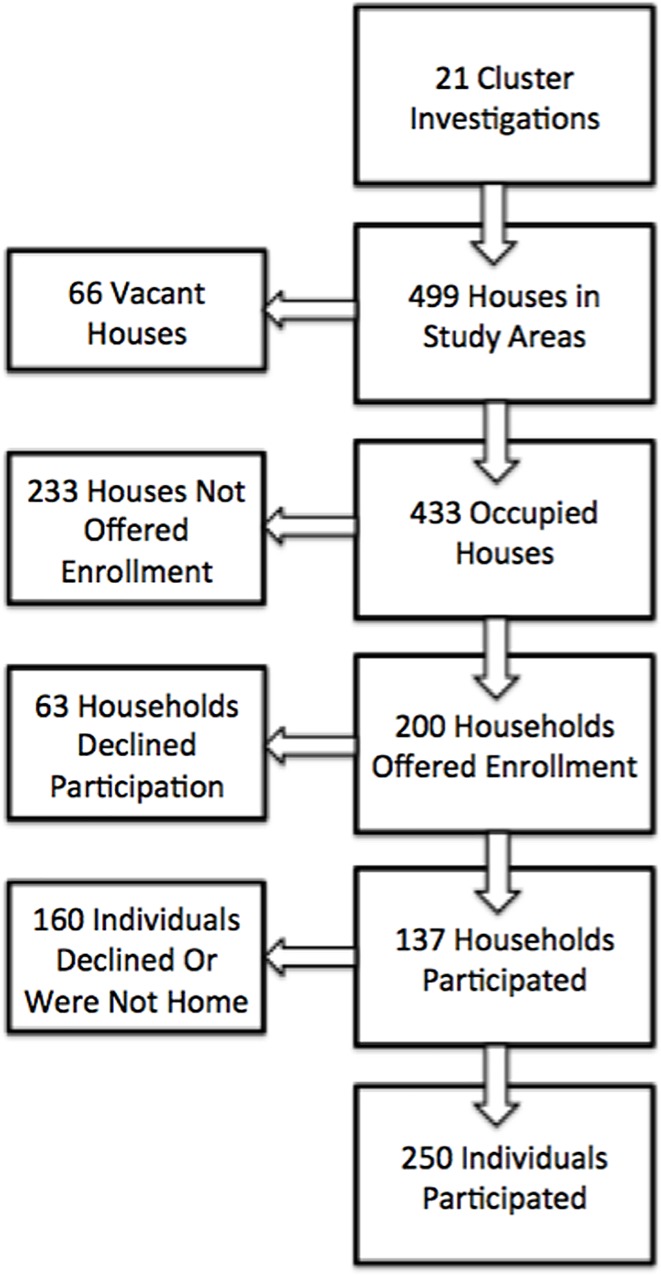
Enrollment characteristics for households and individuals included in chikungunya cluster investigations conducted in Puerto Rico, 2014.

A total of 21 household-based cluster investigations were conducted in the health regions of San Juan, Bayamón, Ponce, Arecibo and Caguas (S1 Table). Of 499 households eligible for participation, heads-of-household from 200 (46.2%) occupied households were available to be offered enrollment, and 137 (68.5%) accepted (Fig 1). Median rate of enrollment by cluster and health region was 66.7% (range: 37.5–100%) and 66.7% (range: 62.5–78.9%), respectively. Of the 410 residents of all enrolled households, 250 (61.0%) participated in the investigation. Participants tended to be older than all residents living in participating households (median age = 45 vs. 25 years, respectively).

**Fig 2:**
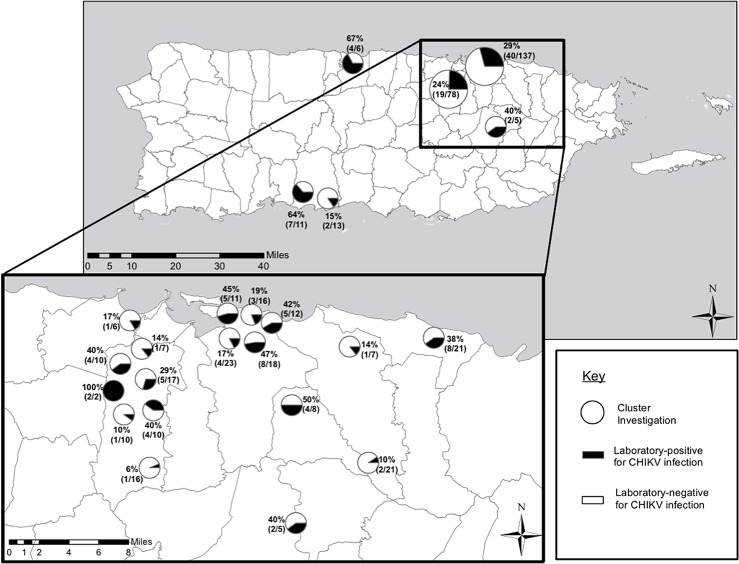
Map depicting chikungunya virus infection rates for each of the 21 household-based cluster investigations conducted in Puerto Rico, 2014.

Of the 250 household cluster investigation participants, 74 (29.6%) had evidence of CHIKV infection. Although infection rates varied by cluster both between and within health regions, all clusters had at least one infected individual apart from the index case-patient (Fig 2). This included 12 participants with current CHIKV infection and 62 participants recent CHIKV infection. Among those with current CHIKV infection, 9 (75.0%) were positive only by rRT-PCR, 1 (8.3%) was positive by rRT-PCR and IgM ELISA, and 2 (16.7%) were positive by rRT-PCR as well as both IgM and IgG ELISA. Of those with recent CHIKV infection, 53 (85.4%) were positive by both IgM and IgG ELISA, 5 (8.1%) were positive by IgM ELISA only, and 4 (6.5%) were positive by IgG ELISA only.

### Duration of detection of CHIKV nucleic acid and anti-CHIKV IgM and IgG antibodies

**Fig 3:**
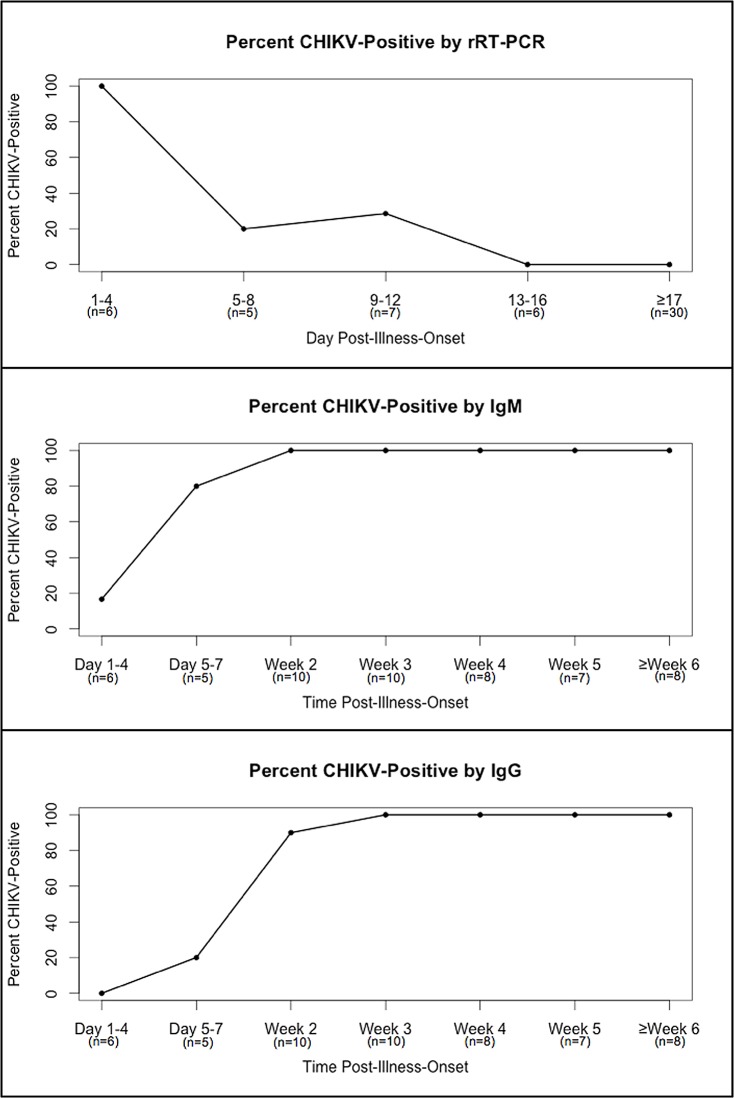
Duration of detection of diagnostic markers of chikungunya virus infection by test and time of specimen collection post-illness-onset.

Duration of detection of diagnostic markers of CHIKV infection was plotted for all participants who had evidence of CHIKV infection by any method and reported recent symptoms of illness and a date of illness onset (n = 54) (Fig 3). All specimens collected before day four post-illness-onset (PIO) were positive by rRT-PCR. Detection of CHIKV nucleic acid by rRT-PCR decreased over time by day of specimen collection PIO, and by day 13 PIO no rRT-PCR-positive specimens were identified. Percent positivity by anti-IgM and IgG ELISA both increased according to day of specimen collection PIO. All specimens collected after week two PIO were IgM-positive, while all specimens collected after week three PIO were IgG-positive.

### Factors associated with CHIKV infection

Following bivariate analysis, age and gender were not significantly associated with CHIKV infection (Table 1). Participants that reported having chronic joint disease or arthritis had nearly two-fold increased odds of having evidence of CHIKV infection. Reporting having had an acute illness in the past three months or having a household member that had an acute illness in the past three months were both associated with 14-fold increased odds of being laboratory-positive for CHIKV infection. No significant associations were found between CHIKV infection and housing type, having screened windows and doors, and reporting leaving doors or windows open regularly. Participants that reported using household air conditioning or citronella candles had two- or three-fold decreased odds of being laboratory-positive for CHIKV infection, respectively.

**Table 1:** Demographics, pre-existing medical conditions, household characteristics, and mosquito avoidance behaviors associated with chikungunya virus infection among household-based cluster investigations participants in Puerto Rico, 2014 (N = 250).

Characteristic, n (%)	Laboratory-positive participants	Laboratory-negative participants	OR (95% CI)	aOR^§^ (95% CI)
	N = 74	N = 176		
Female gender	39 (52.7)	117 (66.5)	0.61 (0.37, 1.03)	**0.59 (0.35, 0.99)**
Age in years, median (range)	46.6 (9–94)	44.6 (1–99)	1.01 (1.00, 1.02)	1.01 (1.00, 1.02)
Years lived in Puerto Rico, median (range)	39.5 (1–94)	34 (1–98)	1.01 (1.00, 1.02)	1.00 (0.99, 1.02)
Traveled outside of Puerto Rico in prior 3 months	4 (5.4)	19 (10.7)	0.39 (0.12, 1.24)	0.42 (0.13, 1.37)
Chronic Medical Conditions				
Diabetes	18 (24.3)	31 (17.6)	1.49 (0.77, 2.88)	1.30 (0.63, 2.65)
Asthma	13 (17.6)	37 (21.0)	0.82 (0.40, 1.67)	1.01 (0.48, 2.12)
Hypertension	30 (40.5)	59 (33.5)	1.27 (0.78, 2.05)	0.94 (0.51, 1.74)
Heart disease	8 (10.8)	16 (9.1)	1.05 (0.43, 2.54)	0.75 (0.29, 1.98)
Joint disease/arthritis	23 (31.1)	32 (18.2)	**1.90 (1.04, 3.47)**	1.92 (0.94, 3.92)
Hypercholesterolemia	23 (31.1)	38 (21.6)	1.36 (0.74, 2.51)	1.08 (0.53, 2.20)
Thyroid disease	14 (18.9)	19 (10.8)	1.71 (0.81, 3.60)	1.79 (0.76, 4.21)
Other*	9 (12.2)	24 (13.6)	0.91 (0.44, 1.88)	0.75 (0.34, 1.64)
Number of chronic medical conditions	1 (0–7)	1 (0–6)	1.11 (0.96, 1.29)	1.07 (0.87, 1.30)
Daily medications				
NSAIDs^†^	9 (12.2)	31 (17.6)	0.67 (0.32, 1.38)	0.46 (0.21, 1.03)
Other^‡^	3 (4.1)	8 (4.6)	0.80 (0.23, 2.83)	0.71 (0.22, 2.37)
Acute illness in the past 3 months	61 (82.4)	38 (21.7)	**14.67 (7.36, 29.23)**	**21.56 (9.24, 50.31)**
Household member with acute illness in the past 3 months	69 (93.2)	87 (50.9)	**14.54 (5.47, 38.63)**	**17.43 (6.19, 49.10)**
Type of home				
One story house	31 (41.9)	69 (40.4)	Ref.	Ref.
Two story house	21 (28.4)	54 (31.6)	1.08 (0.74, 1.59)	0.85 (0.40, 1.83)
Apartment/Condominium	22 (29.7)	48 (28.1)	0.93 (0.45, 1.92)	1.03 (0.49, 2.16)
Home has screened windows and doors				
Yes, all or some rooms	46 (62.2)	98 (57.3)	1.15 (0.61, 2.15)	1.07 (0.57, 2.01)
No	28 (37.8)	73 (42.7)	Ref.	Ref.
Use air conditioning in home regularly				
Yes, in all or some rooms	31 (41.9)	99 (57.9)	**0.48 (0.26, 0.90)**	**0.50 (0.27, 0.94)**
No	43 (58.1)	72 (42.1)	Ref.	Ref.
Leave doors or windows open regularly				
Ever	64 (86.5)	138 (81.2)	1.49 (0.64, 3.47)	1.49 (0.63, 3.53)
Never	10 (13.5)	32 (18.8)	Ref.	Ref.
Use mosquito coils in house or patio	18 (24.3)	50 (29.6)	0.75 (0.36, 1.56)	0.84 (0.40, 1.75)
Use citronella candles in house or patio	7 (9.7)	35 (20.7)	**0.33 (0.12, 0.89)**	**0.37 (0.14, 0.99)**
Used mosquito repellant in the past month	23 (31.1)	60 (34.1)	0.91 (0.51, 1.64)	1.06 (0.58, 1.94)

OR = odds ratio; aOR = adjusted odds ratio; 95% CI = 95% confidence interval

^**§**^Adjusted for age and gender

*Stroke, cancer, kidney disease, lung disease, liver disease

^†^Non-steroidal anti-inflammatory drugs

^‡^Corticosteroids, antibiotics

Following multivariable analysis that controlled for age and gender, female gender was associated with protection from CHIKV infection. Neither reporting having a chronic medical condition nor use of daily medications was associated with protection from CHIKV infection. Reporting having an acute illness or having a household member with an acute illness in the past three months both remained strongly associated with increased odds of CHIKV infection. Use of mosquito repellent and citronella candles remained associated with protection from CHIKV infection.

### Signs and symptoms associated with chikungunya virus infection

**Fig 4:**
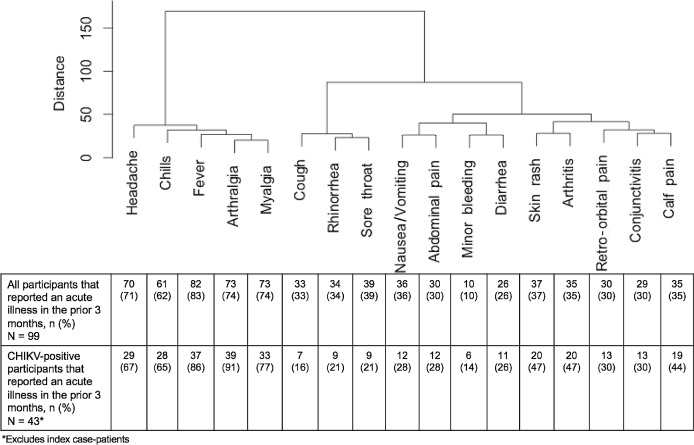
Dendrogram of hierarchical cluster analysis of symptoms among household-based cluster investigation participants that reported an acute illness in the previous three months, Puerto Rico, 2014

Of 99 participants that reported having an acute illness within the previous three months, 61 (61.6%) were laboratory-positive for CHIKV infection (Table 2). Median duration of illness in laboratory-positive participants was six days (range: 2–21 days). Following bivariate analysis, signs and symptoms associated with CHIKV infection in ill participants were fever, skin rash, arthralgia, and arthritis. Cough, rhinorrhea, and sore throat were associated with being laboratory-negative for CHIKV infection. No laboratory-positive symptomatic participants reported cough, rhinorrhea, or sore throat in the absence of fever or arthralgia. Following multivariable analysis, arthralgia and skin rash remained significantly associated with laboratory-positive symptomatic participants, and only retro-orbital eye pain remained significantly associated with laboratory-negative symptomatic participants. Headache, fever, arthralgia, myalgia, and chills tended to occur simultaneously more often among laboratory-positive participants, whereas cough, rhinorrhea, and sore throat occurred together more often among laboratory-negative participants (Fig 4).

**Table 2:** Illness and health care seeking behaviors of household-based cluster investigation participants that reported an acute illness in the previous three months, Puerto Rico, 2014.

Characteristic, n (%)	Laboratory-positive participants	Laboratory-negative participants	OR (95% CI)	aOR^§^ (95% CI)
	N = 61	N = 38		
Days from illness onset until specimen collection, median (range)	19 (1–60)	17.5 (1–75)	0.97 (0.94, 1.00)	—
Duration of illness (days), median (range)	6 (2–21)	7 (1–45)	0.95 (0.90, 1.01)	—
Symptoms of illness				
Fever	55 (90.2)	27 (71.1)	**3.73 (1.37, 20.15)**	6.32 (0.45, 89.36)
Chills	42 (68.9)	19 (50.0)	2.29 (0.91, 5.30)	—
Nausea/vomiting	21 (34.4)	15 (39.5)	0.80 (0.39, 1.61)	—
Diarrhea	17 (27.9)	9 (23.7)	1.33 (0.58, 3.02)	—
Myalgia	49 (80.3)	24 (63.2)	2.38 (0.94, 6.04)	0.07 (0.00, 1.17)
Arthralgia	57 (93.4)	16 (42.1)	**19.01 (6.60, 54.76)**	**51.81 (3.83, 700.82)**
Skin rash	32 (52.5)	5 (13.2)	**6.97 (2.39, 20.35)**	**14.28 (2.41, 84.72)**
Conjunctivitis	20 (32.8)	9 (23.7)	1.64 (0.63, 4.27)	—
Headache	42 (68.9)	28 (73.7)	0.82 (0.35, 1.93)	3.49 (0.80, 15.20)
Retro-orbital eye pain	18 (29.5)	12 (31.6)	0.93 (0.37, 2.35)	**0.07 (0.01, 0.52)**
Abdominal pain	18 (29.5)	12 (31.6)	0.89 (0.41, 1.91)	—
Cough	13 (21.3)	20 (52.6)	**0.23 (0.09, 0.61)**	—
Rhinorrhea	13 (21.3)	21 (55.3)	**0.21 (0.10, 0.48)**	0.55 (0.14, 2.12)
Sore throat	15 (24.6)	24 (63.2)	**0.19 (0.07, 0.51)**	0.22 (0.05, 1.02)
Calf pain	26 (42.6)	9 (23.7)	2.41 (0.97, 5.97)	—
Arthritis	30 (49.2)	5 (13.2)	**6.27 (2.30, 17.07)**	—
Minor bleeding*	7 (11.5)	3 (7.9)	1.45 (0.31, 6.68)	—
Major bleeding^†^	0	2 (5.3)	NA	—
Sought medical care^‡^	27 (64.3)	13 (35.1)	**3.30 (1.31, 8.31)**	—
Diagnosis				—
Chikungunya	5 (18.5)	0	NA	—
Dengue	2 (7.4)	0	NA	—
Viral syndrome	10 (37.0)	2 (15.4)	3.03 (0.56, 16.42)	—
Unknown	4 (14.8)	2 (15.4)	0.89 (0.14, 5.52)	—
Other (unspecified)	8 (29.6)	9 (69.2)	**0.19 (0.04, 0.84)**	—
Hospitalized due to Illness	3 (11.5)	2 (15,4)	0.67 (0.10, 4.57)	—
Length of hospital stay (days), median (range)	4 (3–7)	7.5 (7–8)	—	—

^§^Adjusted OR and 95% CI are shown for covariates that were included in the best-fitting multivariate GEE model

*Petechia, gingival bleeding, epistaxis, unexplained bruising

^†^Hematemesis, hemoptysis, melena, menorrhagia

^‡^Excludes index case-patients

NA = not applicable, since the GEE model was unable to produce an odds ratio due to zero variance for at least one comparison group as a positive definite covariance matrix is required to produce estimates

### Definition of symptomatic CHIKV infection

Because of the prevalence of respiratory illness concurrent with chikungunya virus transmission, combinations of symptoms that grouped together following hierarchical cluster analysis and were most frequently reported among laboratory-positive participants following multivariate analysis were utilized to refine the definition of “symptomatic CHIKV infection” in order to minimize incorrect classification of symptomatically-infected participants (S2 Table). The maximal association of symptom combinations among laboratory-positive participants with concomitant minimization of association with laboratory-negative participants was arthralgia with skin rash or fever. This combination of symptoms yielded a symptomatic CHIKV infection rate of 62.5%, and was present among 6.8% of participants without evidence of CHIKV infection. This combination of symptoms was utilized in subsequent analyses to define “symptomatic CHIKV infection”.

### Factors associated with asymptomatic CHIKV infection

Twenty-one (37.5%) participants, including two that had CHIKV nucleic acid detected by RT-PCR, were defined as having asymptomatic infection. Age was not significantly associated with asymptomatic infection (Table 3), nor was being a child (1 of 5 [20%] children with asymptomatic infection vs. 20 of 51 [39%] adults; OR = 0.30, 95% CI = 0.03–3.67). Neither sex nor reported chronic medical conditions was significantly associated with asymptomatic infection. Participants who reported having a household member with an acute illness within the previous three months more often had symptomatic infection (100% vs. 81%).

**Table 3:** Characteristics associated with asymptomatic chikungunya virus infection among participants of household-based cluster investigations conducted in Puerto Rico, 2014.

Characteristic	Asymptomatic	Symptomatic	OR (95% CI)
	N = 21	N = 35	
Female gender, n (%)	12 (57.1)	19 (54.3)	1.19 (0.41, 3.40)
Age in years, median (range)	54.1 (9.6–83.4)	45.2 (9.9–94.0)	1.00 (0.98, 1.03)
Chronic medical conditions			
Diabetes	8 (38.1)	7 (20.0)	2.23 (0.65, 7.60)
Asthma	2 (9.5)	6 (17.1)	0.46 (0.08, 2.56)
Hypertension	10 (47.6)	16 (45.7)	1.01 (0.33, 3.10)
Heart disease	3 (14.3)	4 (11.4)	1.78 (0.40, 7.89)
Joint disease/arthritis	9 (42.9)	8 (22.9)	2.27 (0.70, 7.37)
Hypercholesterolemia	9 (42.9)	12 (34.3)	1.17 (0.39, 3.52)
Thyroid disease	5 (23.8)	5 (14.3)	1.70 (0.43, 6.71)
Other*	2 (9.5)	5 (14.3)	0.60 (0.11, 3.40)
Taking NSAIDs^†^	2 (9.5)	5 (14.3)	0.60 (0.11, 3.40)
Ill household member in previous 3 months	17 (81.0)	35 (100)	NA

*Kidney disease, lung disease, stroke

^†^Nonsteroidal anti-inflammatory drugs

NA = not applicable, since the GEE model was unable to produce an odds ratio due to zero variance for at least one comparison group as a positive definite covariance matrix is required to produce estimates

### Factors associated with seeking medical care, clinical diagnosis, and case reporting

After again excluding the index case-patients, 22 (62.9%) of 35 symptomatic, laboratory-positive participants sought medical care. Seeking medical care for acute illness was associated with 3-fold increased odds of being laboratory-positive (Table 2). Neither hospitalization nor duration of illness was significantly associated with being laboratory-positive for CHIKV infection. No demographic or clinical characteristics were significantly associated with seeking medical care.

Of 22 laboratory-positive, symptomatic participants that sought medical care, five (22.7%) reported having been diagnosed with chikungunya (S3 Table). Neither age nor sex were significantly associated with correct reported diagnosis of chikungunya. All laboratory-positive, symptomatic patients diagnosed with chikungunya reporting having arthralgia in the hands, wrist, knee, ankle, and feet. Two (9.1%) laboratory-positive, symptomatic participants that sought medical care were reported to public health authorities.

## Discussion

By conducting household-based cluster investigations during the early months of the 2014 chikungunya epidemic in Puerto Rico that included 250 participants residing within 50 meters of a known chikungunya case-patient, we found that 30% of participants had evidence of CHIKV infection. Reporting having had an acute illness in the past three months and having a household member with an acute illness were associated with increased odds of infection, while use of either air conditioning or citronella candles were associated with decreased odds of infection. Symptoms significantly associated with CHIKV infection included arthralgia and skin rash. Nearly two-thirds of symptomatically-infected individuals sought medical care; however, less than one-quarter of these individuals were diagnosed with chikungunya, and one-tenth were reported to public health authorities as a chikungunya case. These findings demonstrate the utility of household-based cluster investigation to describe the epidemiologic and clinical characteristics associated with an emerging infectious disease and reasons for underreporting of clinically-apparent disease cases.

Serosurveys following chikungunya epidemics in Malaysia, Kenya, La Reunion, and Mayotte Island reported infection rates ranging from 37–75% [2–5]. Overall, 30% of participants in this investigation had evidence of CHIKV infection, which varied by cluster from 6.3% to 100%. These estimates likely do not reflect the final infection rates in these communities, as investigations were conducted during the first weeks of the epidemic in Puerto Rico where further CHIKV transmission likely occurred. A critical facet regarding interpretation of these results is that the objective of this investigation was not to determine the number of individuals infected with CHIKV during the indicated time frame, but rather to identify and compare the behaviors and characteristics of infected and uninfected participants. As such, the estimates of seroprevalence from previous studies and our findings are not directly comparable, as previous studies retrospectively measured rate of infection whereas this investigation collected a cross-sectional “snapshot” of infection rates during the initial stages of the epidemic.

Nonetheless, demographic and behavioral characteristics were able to be associated with susceptibility to or protection from CHIKV infection. Having a household member with acute illness in the last three months was strongly associated with increased the odds of infection, which supports the notion that, like DENV, CHIKV infections tend to cluster within households and neighborhoods [30]. Air conditioning use was associated with decreased odds of CHIKV infection, as has been reported in previous studies of DENV infection [31]. This finding may not be attributable to cooler temperatures in air conditioned homes but rather to buildings with air conditioning tending to have closed windows and doors and drier environments that result in lower rates of survival of *Ae*. *aegypti* mosquitos [32]. Use of citronella candles was also associated with reduced odds of CHIKV infection; however, the proportion of all participants using citronella candles was relatively small (17%), and thus likely did not contribute substantially to protection from infection on a population level. Past studies have shown varying and inconsistent levels of reduction of mosquito abundance associated with citronella candles [33, 34] as the quantity, concentration, and positioning of candles may play a role in their effectiveness [35].

By using findings from multivariable and hierarchical cluster analyses to identify arthralgia with fever or rash as being associated with CHIKV infection, we were able to more confidently define the rate of symptomatic CHIKV infection in this population as being 62.5%. Conversely, one-third of CHIKV-infected participants appear to have experienced asymptomatic infection, which is consistent with findings from past outbreaks that reported asymptomatic infection rates of 3–39% [36–38]; however, recent reports have suggested substantially higher rates of asymptomatic infection (e.g., 81%) [39]., Hence, further investigation including careful and potentially region-specific definitions of symptomatic infection is needed to determine factors influencing the rate of and progression to symptomatic CHIKV infection among diverse populations.

Most CHIKV-infected participants identified in this investigation that reported an acute illness in the past three months complained of characteristic symptoms of chikungunya: fever, arthralgia, myalgia, and skin rash [6]. Laboratory-negative CHIKV participants with recent illness were more likely to report symptoms of cough, rhinorrhea, or sore throat, suggestive of an upper respiratory infection. Symptomatic laboratory-positive CHIKV participants had three-fold increased odds of having sought medical care compared to participants that were laboratory-negative with reported recent illness. These observations together suggest greater disease severity of chikungunya as compared to common respiratory illnesses. Future studies should quantitate the burden of the chikungunya epidemic on health care resources in Puerto Rico.

Nearly two-thirds of symptomatically-infected patients sought medical care, demonstrating a relatively high rate of care-seeking behavior that may reflect the increased severity of arthralgia and myalgia as compared to patients with other etiologies of acute febrile illness. However, only one-quarter of chikungunya patients that sought care reported having been diagnosed with chikungunya, suggesting gaps in clinical suspicion of chikungunya. Other common diagnoses included more common etiologies of acute febrile illness including dengue and non-specific diagnoses such as viral syndrome. Because just one-tenth of clinically apparent chikungunya cases were reported as such to public health authorities, it is unclear how accurately the number of chikungunya cases reported to PRDH in 2014 reflects the true incidence of disease due to CHIKV infection. As with other reportable conditions for which passive case reporting is sub-optimal [40], including dengue [41, 42], the identified gaps in case detection via passive surveillance should be taken into consideration when making estimates of the burden of symptomatic and clinically-apparent chikungunya.

Strengths of this investigation included the ability to detect asymptomatic, sub-clinical, and clinically-apparent CHIKV infections, as well as the use of three different laboratory tests to identify current or recent CHIKV infection. It is therefore unlikely that any participants with CHIKV infection were not identified. Similarly, recall bias was likely to have been minimal since questionnaires captured events that had occurred within the prior three months. Conversely, a convenience sample of reported chikungunya cases was utilized to initiate cluster investigations, most of which were conducted in the San Juan metropolitan area. Moreover, factors that can influence both mosquito density (e.g., rainfall, temperature, humidity) [43] as well as the efficiency of CHIKV transmission (e.g., population density) [44] vary throughout Puerto Rico. For both of these reasons, our findings may not be representative of the entire population of Puerto Rico. Last, four laboratory-positive participants were defined as such by detection of anti-CHIKV IgG antibody only. Because lifetime travel history was not captured, it is possible that these individuals had been infected with CHIKV outside of Puerto Rico. Nonetheless, exclusion of these four individuals would not have significantly altered the observed associations.

Factors identified with protection from CHIKV infection identified in this investigation were household rather than individual behaviors, suggesting the importance of prevention practices in and around the household. Such behaviors should be encouraged in areas where *Aedes* mosquitoes are found. The clinical findings of this investigation highlight the need for increased capacity to identify chikungunya patients in out-patient settings. Due to the difficulty in utilizing signs and symptoms alone to differentiate patients with chikungunya from other febrile illnesses, clinical diagnosis and decision-making as well as case reporting would be aided by improved availability of rapid diagnostic tests.

## Supporting Information

S1 Checklist STROBE Checklist (DOCX)

S1 Table Characteristics of households and individuals included in chikungunya cluster investigations conducted in Puerto Rico, 2014 (N = 21). (DOCX)

S2 Table Signs and symptoms of recent illness reported by participants of chikungunya virus household-based cluster investigations conducted in Puerto Rico, 2014 (N = 232). (DOCX)

S3 Table Characteristics associated with diagnoses of laboratory-positive, symptomatic participants of household cluster investigations that sought medical care, Puerto Rico, 2014 (N = 22). (DOCX)

S1 Fig Timing of when household cluster investigations were conducted relative to the temporality of the 2014 chikungunya epidemic in Puerto Rico. (TIF)

S1 Appendix Chikungunya household investigation–Household Questionnaire. (PDF)

S2 Appendix Chikungunya household investigation–Individual Questionnaire. (PDF)

S1 Dataset Case control investigation data (XLSX)
