# Supplementary material for: Use of Household Cluster Investigations to Identify Factors Associated with Chikungunya Virus Infection and Frequency of Case Reporting in Puerto Rico
Source: PLoS Negl Trop Dis. 2016 Oct 20;10(10):e0005075. doi: 10.1371/journal.pntd.0005075 (PMC5072658; doi:10.1371/journal.pntd.0005075)
Supplement: S1 Table — (DOCX) [file pntd.0005075.s002.docx]

**S1 Table. Characteristics of households and individuals included in chikungunya cluster investigations conducted in Puerto Rico, 2014 (N = 21).**

| **Characteristic, median (range)** | **San Juan**  **(n = 9)** | **Bayamón**  **(n = 8)** | **Ponce**  **(n = 2)** | **Arecibo**  **(n = 1)** | **Caguas**  **(n = 1)** | **ALL**  **(N = 21)** |
| --- | --- | --- | --- | --- | --- | --- |
| Days from index case-patient’s illness onset to investigation | 22.5 (12 – 42) | 35 (12 – 42) | 32.5 (32 – 33) | 32 | 48 | 32 (12 – 48) |
| Households per cluster, median (range) | 29 (12 – 40) | 20 (4 – 30) | 30.5 (30 – 31) | 4 | 16 | 26 (4 – 40) |
| Occupied households per cluster, % (range) | 91.7 (63.6 – 100) | 89.7 (84.6–100) | 67.2 (63.3–71.0) | 75.0 | 100 | 89.7 (63.3–100) |
| Households offered enrollment, % (range) | 50.0 (26.7 – 63.6) | 51.5 (13.3–100) | 46.8 (40.9–52.6) | 100 | 37.5 | 50.0 (13.3–100) |
| Households that participated, % (range) | 66.7 (61.1 – 100) | 64.1 (37.5–100) | 79.4 (70.0–88.9) | 66.7 | 66.7 | 66.7 (37.5–100) |
| Participants per household, median (range) | 1 (1 – 5) | 1 (1 – 6) | 1 (1 – 3) | 3 (2 – 4) | 1 (1 – 2) | 1 (1 – 6) |
| Participants per cluster, median (range) | 16 (7 – 23) | 10 (2 – 17) | 12 (11 – 13) | 6 | 5 | 11 (2 – 23) |
| **Individuals who agreed to participate, N** | **137** | **78** | **24** | **6** | **5** | **250** |
| Laboratory-positive participants, n (%) | 40 (29.2) | 19 (24.4) | 9 (37.5) | 4 (66.7) | 2 (40.0) | 74 (29.6) |
