# Supplementary material for: Use of Household Cluster Investigations to Identify Factors Associated with Chikungunya Virus Infection and Frequency of Case Reporting in Puerto Rico
Source: PLoS Negl Trop Dis. 2016 Oct 20;10(10):e0005075. doi: 10.1371/journal.pntd.0005075 (PMC5072658; doi:10.1371/journal.pntd.0005075)
Supplement: S2 Table — (DOCX) [file pntd.0005075.s003.docx]

**S2 Table. Signs and symptoms of recent illness reported by participants of chikungunya virus household-based cluster investigations conducted in Puerto Rico, 2014 (N = 232).**

| **Symptoms** | **Laboratory-positive Participants***  **(N = 56)** | **Laboratory-negative Participants**  **(N =176 )** |
| --- | --- | --- |
| Any | 43 (76.8) | 38 (21.6) |
| Arthralgia | 39 (69.6) | 16 (9.1) |
| Rash | 20 (35.7) | 5 (2.8) |
| Fever | 37 (66.1) | 27 (15.3) |
| Arthralgia OR Fever | 41 (73.2) | 31 (17.6) |
| Arthralgia OR Rash | 30 (53.6) | 17 (9.7) |
| Arthralgia OR Fever OR Rash | 41 (73.2) | 32 (18.2) |
| Arthralgia AND Fever | 35 (62.5) | 12 (6.8) |
| Arthralgia AND Rash | 19 (33.9) | 4 (2.3) |
| Fever AND Rash | 20 (35.7) | 4 (2.3) |
| Arthralgia AND Fever AND Rash | 19 (33.9) | 4 (2.3) |
| **Arthralgia AND Fever OR Rash** | **35 (62.5)** | **12 (6.8)** |
| Fever AND Arthralgia OR Rash | 36 (64.3) | 12 (6.8) |
| Rash AND Fever OR Arthralgia | 20 (35.7) | 5 (2.8) |
| Fever OR Arthralgia OR Rash, NO rhinorrhea | 32 (57.1) | 15 (8.5) |
| Fever OR Arthralgia OR Rash, NO retro-orbital eye pain | 28 (50.0) | 21 (11.9) |

*excluding index case-patients
