# Supplementary material for: Use of Household Cluster Investigations to Identify Factors Associated with Chikungunya Virus Infection and Frequency of Case Reporting in Puerto Rico
Source: PLoS Negl Trop Dis. 2016 Oct 20;10(10):e0005075. doi: 10.1371/journal.pntd.0005075 (PMC5072658; doi:10.1371/journal.pntd.0005075)
Supplement: S3 Table — (DOCX) [file pntd.0005075.s004.docx]

**S3 Table. Characteristics associated with diagnoses of laboratory-positive, symptomatic participants of household cluster investigations that sought medical care, Puerto Rico, 2014 (N = 22).**

| **Characteristic** | **Diagnosed with Chikungunya**  ( **N = 5)** | **Not Diagnosed with Chikungunya**  **(N = 17)** | **OR (95%CI)** |
| --- | --- | --- | --- |
| **Age in years, median (range)** | 66.0 (22.7–78.4) | 41.7 (9.9–94.0) | 1.02 (0.98, 1.08) |
| **Female gender, n (%)** | 3 (60.0) | 10 (58.8) | 1.09 (0.16, 7.33) |
| **Signs and symptoms, n (%)** |  |  |  |
| Fever | 5 (100) | 17 (100) | NA |
| Chills | 3 (60.0) | 15 (88.2) | 0.21 (0.02, 2.15) |
| Nausea | 3 (60.0) | 4 (23.5) | 4.61 (0.63, 33.8) |
| Arthralgia | 5 (100) | 17 (100) | NA |
| Hand | 5 (100) | 12 (70.6) | NA |
| Wrist | 5 (100) | 12 (70.6) | NA |
| Shoulder | 4 (80.0) | 11 (64.7) | 1.98 (0.20, 19.65) |
| Knee | 5 (100) | 16 (94.1) | NA |
| Ankle | 5 (100) | 15 (88.2) | NA |
| Foot | 5 (100) | 11 (64.7) | NA |
| Skin rash | 3 (60.0) | 9 (52.9) | 1.30 (0.22, 7.68) |
| Conjunctivitis | 3 (60.0) | 6 (35.3) | 2.87 (0.35, 23.27) |
| Headache | 3 (60.0) | 12 (70.6) | 0.67 (0.08, 5.42) |
| Retro-orbital pain | 5 (100) | 3 (17.7) | NA |
| Abdominal pain | 3 (60.0) | 3 (17.7) | 6.55 (0.84, 60.82) |
| Cough | 2 (40.0) | 3 (17.7) | 2.96 (0.33, 26.55) |
| Rhinorrhea | 2 (40.0) | 4 (23.5) | 2.10 (0.29, 15.07) |
| Sore throat | 1 (20.0) | 2 (11.8) | 2.40 (0.12, 25.09) |
| Calf pain | 3 (60.0) | 8 (47.1) | 1.79 (0.22, 13.39) |
| Arthritis | 5 (100) | 8 (47.1) | NA |
| Minor bleeding | 0 (0) | 3 (17.7) | NA |
| Muscle pain | 5 (100) | 13 (76.5) | NA |
| Diarrhea | 3 (60.0) | 6 (35.3) | 2.60 (0.33, 20.64) |
| **Number of comorbid conditions, median (range)** | 2 (0–6) | 1 (0–7) | 1.04 (0.68, 1.58) |
| **Ill household member in previous three months, n (%)** | 5 (100) | 17 (100) | NA |

NA = not applicable, since the GEE model was unable to produce an odds ratio due to zero variance for at least one comparison group as a positive definite covariance matrix is required to produce estimates
