# Supplementary figures and images for: Use of Household Cluster Investigations to Identify Factors Associated with Chikungunya Virus Infection and Frequency of Case Reporting in Puerto Rico
Source: PLoS Negl Trop Dis. 2016 Oct 20;10(10):e0005075. doi: 10.1371/journal.pntd.0005075 (PMC5072658; doi:10.1371/journal.pntd.0005075)

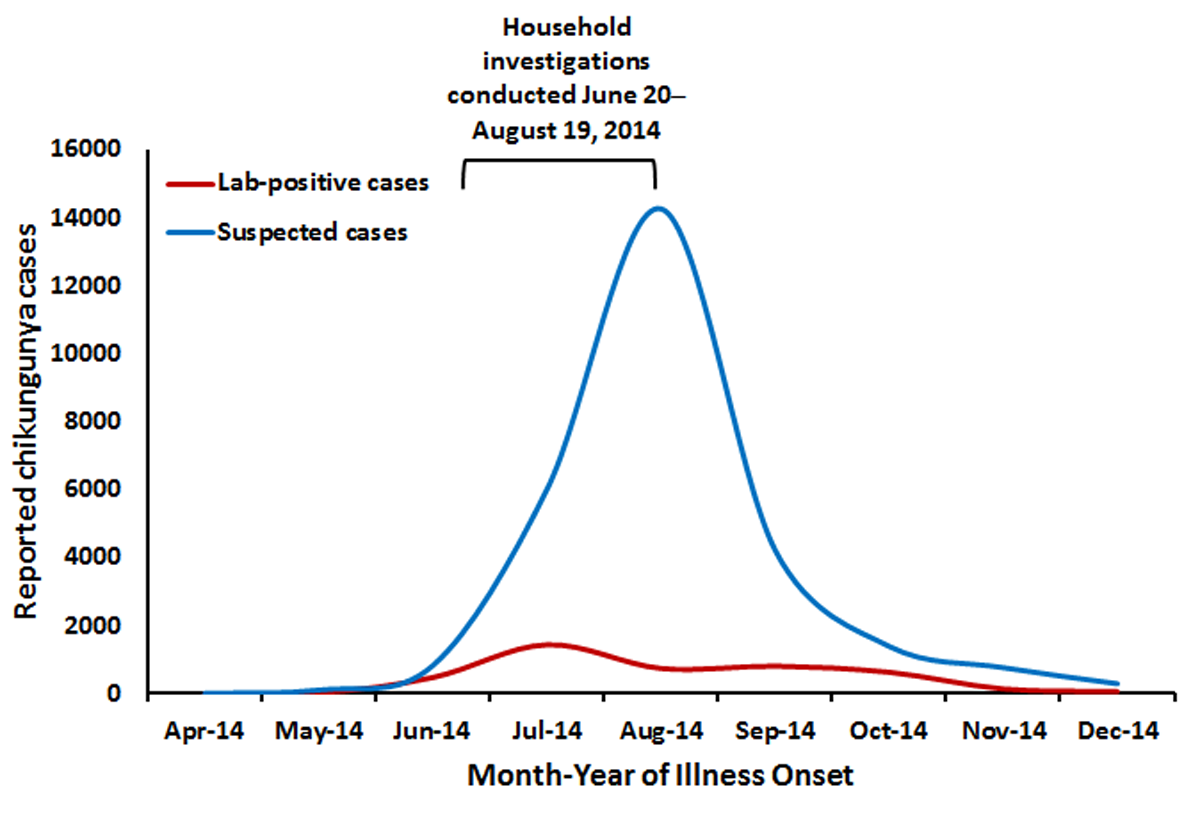

Supplement: S1 Fig — (TIF) [file pntd.0005075.s005.tif]
