# Supplementary material for: Use of Household Cluster Investigations to Identify Factors Associated with Chikungunya Virus Infection and Frequency of Case Reporting in Puerto Rico
Source: PLoS Negl Trop Dis. 2016 Oct 20;10(10):e0005075. doi: 10.1371/journal.pntd.0005075 (PMC5072658; doi:10.1371/journal.pntd.0005075)
Supplement: S1 Appendix — (PDF) [file pntd.0005075.s006.pdf]

CHIKUNGUNYA INVESTIGATION — HOUSEHOLD INTERVIEW FORM

TEAM #: ________________ DATE: ______/______/_________ Household ID (e.g., SJ-1-A): _______-______-_____

GPS Coordinates: _____._____________ , ______._______________ SANID of lab-positive case: _____________________

How many people live in this house? ________________people
List all members of household below put yourself first.

Head of household contact number to facilitate return of test results:____

	**Name** (First, Paternal, Maternal)	**Age**	**Gender**	**Participate?**	**Place sticker here**
1			M / F	Yes / No	
2			M / F	Yes / No	
3			M / F	Yes / No	
4			M / F	Yes / No	
5			M / F	Yes / No	
6			M / F	Yes / No	
7			M / F	Yes / No	
8			M / F	Yes / No	

CHIKUNGUNYA INVESTIGATION — HOUSEHOLD INTERVIEW FORM

Household Characteristics

Housing type (check only one): ☐ One story house ☐ Two story house ☐ Apartment/condo building

☐Public housing ☐ Temporary shelter

Has anyone in your immediate household traveled outside of Puerto Rico in the past 3 months? ☐ Yes ☐ No

Has anyone in your household been sick in the past 3 months? ☐ Yes ☐ No

Does your home have screened windows and doors? ☐ All rooms ☐ Some rooms ☐ No

Do you regularly use air conditioning in your home? ☐ Yes, in all rooms ☐ Yes, but only in some rooms ☐ No

Do you regularly leave your doors or windows open? ☐ Daytime only ☐ Night-time only ☐ Always ☐ Never

Do you use mosquito coils in your house or patio? ☐ Yes ☐ No

Do you use citronela in your house or patio to keep mosquitoes away? ☐ Yes ☐ No

Notes:
