# Supplementary material for: Use of Household Cluster Investigations to Identify Factors Associated with Chikungunya Virus Infection and Frequency of Case Reporting in Puerto Rico
Source: PLoS Negl Trop Dis. 2016 Oct 20;10(10):e0005075. doi: 10.1371/journal.pntd.0005075 (PMC5072658; doi:10.1371/journal.pntd.0005075)
Supplement: S2 Appendix — (PDF) [file pntd.0005075.s007.pdf]

# CHIKUNGUNYA INVESTIGATION — INDIVIDUAL INTERVIEW FORM

Team #: _______ Interviewer: _________ Date of interview: _____/_____/________

Individual ID (e.g., SJ-1-A-1): ______-_____-_____-____

1. Name: ____________________________________________________________
First (given) Initial Paternal Maternal

2. Gender: ☐ Male ☐Female 3. Date of Birth (MM/DD/YYYY): ____/_____/______

4. How long have you been living in Puerto Rico? _______ years

5. Have you been told by a clinician that you have any of the following medical conditions?

☐ Diabetes ☐ High blood pressure ☐ Heart disease ☐ High cholesterol

☐ Stroke ☐ Kidney disease ☐ Liver disease ☐ Thyroid disease

☐ Asthma ☐ Lung disease ☐ Joint disease/arthritis ☐ Cancer

6. Do you take any of the following medications daily:

☐ NSAID (e.g., aspirin, Iburpofen) ☐ Corticosteroids ☐ Antibiotics

7. Have you experiencing any new illnesses in the past 3 months? ☐Yes ☐ No
*(If more than one illness episode, detail each additional episode in Notes.)*

7a. If yes, first day of illness (MM/DD/YYYY): ____/_______/_____________

7b. What symptoms did you have (check all that apply)?

☐ Fever ☐ Chills ☐ Nausea/Vomiting ☐ Diarrhea

☐ Muscle pain ☐ Joint pain ☐ Skin rash ☐ Red eyes

☐ Headache ☐ Pain behind eyes ☐ Abdominal pain ☐ Cough

☐ Runny nose ☐ Sore throat ☐ Calf pain ☐ Arthritis (red, swollen joints)

☐ Minor bleeding (e.g., petechia, gum bleed, nosebleed, severe bruising)

☐ Major bleeding (e.g., vomiting blood, coughing up blood, blood in stool, heavy menses)

7b-1. If you had joint pain, indicate the locations where you had the pain

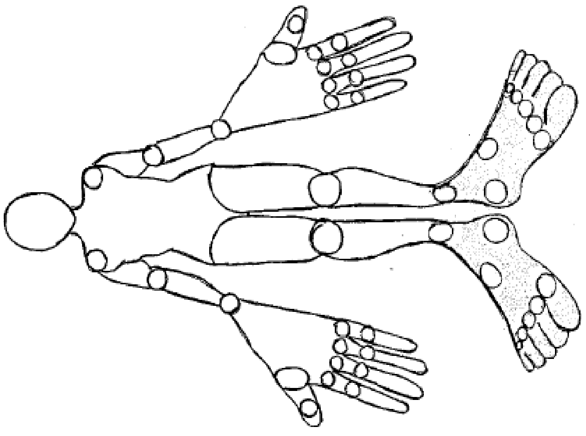

# CHIKUNGUNYA INVESTIGATION — INDIVIDUAL INTERVIEW FORM

7c. How long did this illness last? ______ days

7d. Did you go to the doctor because of this illness? ☐ Yes ☐ No

7d-1. If yes, Name of hospital/clinic:________________________________________

7d-2. What was the diagnosis? ☐Chikungunya ☐ Dengue

☐ Viral syndrome ☐ I don't know ☐ Other: _________________________

7d-3. Were you hospitalized for this illness? ☐ Yes ☐ No

7d-3a. If yes, Hospital Name: _________________________________

7d-3b. Days in the hospital: _____ days

8. Have you used mosquito repellent in the past month? ☐ Daily ☐ Weekly ☐ Never

9. Have you slept under a bednet in the past month? ☐ Yes ☐ No

10. Have you traveled outside of Puerto Rico in the past 3 months? ☐ Yes ☐ No

10a. If yes, specify where and date of return to Puerto Rico for the most recent trip:

☐ United States (excluding USVI) ☐ Dominican Republic ☐Caribbean cruise

☐Other: ______________________________________________________________

Date of return to PR (MM/DD/YYYY): _______/______/_____________

**NOTES**:
